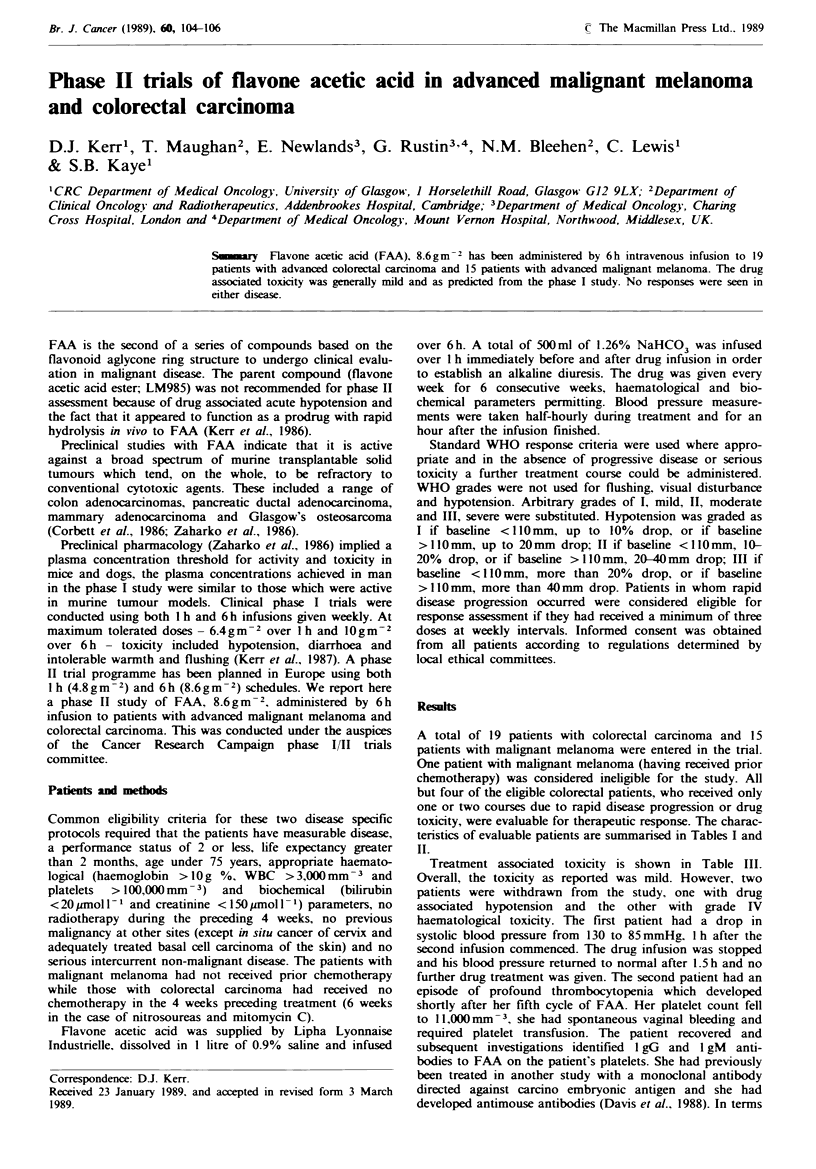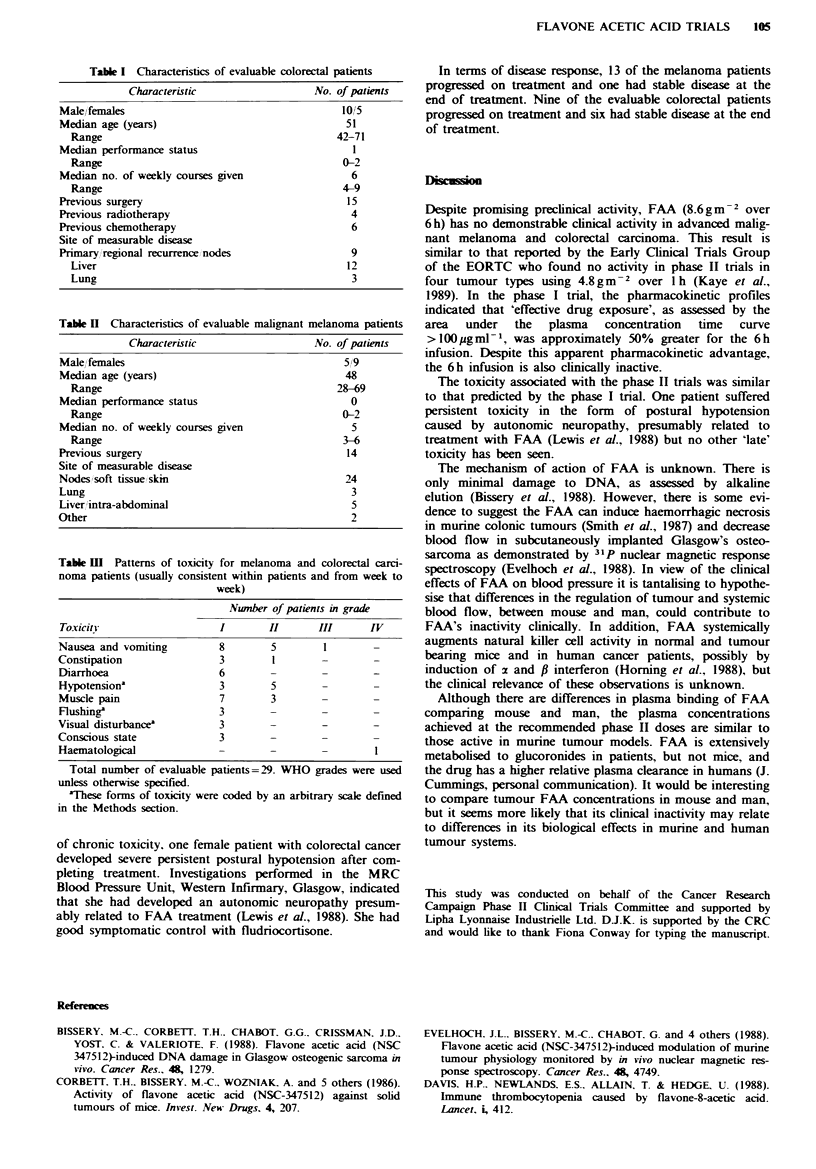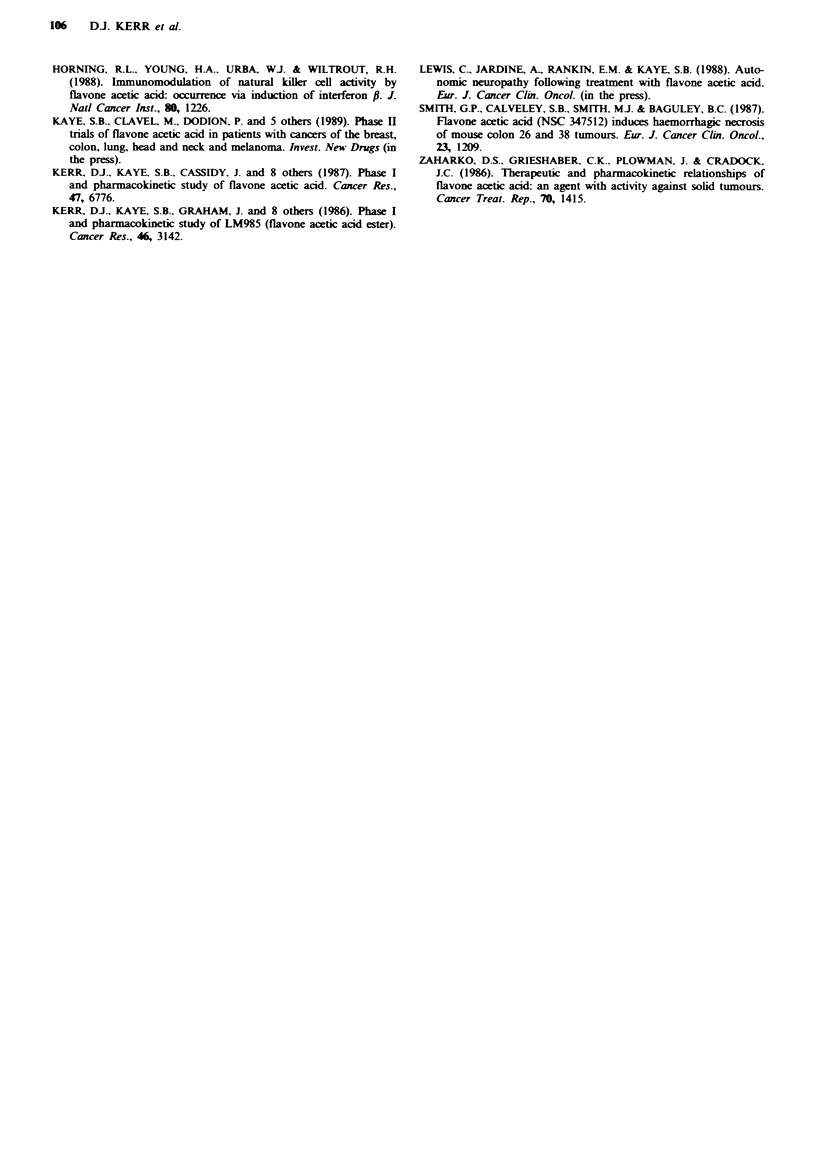# Phase II trials of flavone acetic acid in advanced malignant melanoma and colorectal carcinoma.

**DOI:** 10.1038/bjc.1989.230

**Published:** 1989-07

**Authors:** D. J. Kerr, T. Maughan, E. Newlands, G. Rustin, N. M. Bleehen, C. Lewis, S. B. Kaye

**Affiliations:** CRC Department of Medical Oncology, University of Glasgow, UK.

## Abstract

Flavone acetic acid (FAA), 8.6 gm-2 has been administered by 6h intravenous infusion to 19 patients with advanced colorectal carcinoma and 15 patients with advanced malignant melanoma. The drug associated toxicity was generally mild and as predicted from the phase I study. No responses were seen in either disease.


Br. J. Cancer (1989), 60, 104–106 © The Macmillan Press Ltd., 1989

### Phase II trials of flavone acetic acid in advanced malignant melanoma and colorectal carcinoma

D.J. Kerr[1], T. Maughan[2], E. Newlands[3], G. Rustin[3,4], N.M. Bleehen[2], C. Lewis[1] & S.B. Kaye[1]

[1]*CRC Department of Medical Oncology, University of Glasgow, 1 Horselethill Road, Glasgow G12 9LX;* [2]*Department of Clinical Oncology and Radiotherapeutics, Addenbrookes Hospital, Cambridge;* [3]*Department of Medical Oncology, Charing Cross Hospital, London and* [4]*Department of Medical Oncology, Mount Vernon Hospital, Northwood, Middlesex, UK.*

**Summary** Flavone acetic acid (FAA), $8.6\,g\,m^{-2}$ has been administered by 6h intravenous infusion to 19 patients with advanced colorectal carcinoma and 15 patients with advanced malignant melanoma. The drug associated toxicity was generally mild and as predicted from the phase I study. No responses were seen in either disease.

FAA is the second of a series of compounds based on the flavonoid aglycone ring structure to undergo clinical evaluation in malignant disease. The parent compound (flavone acetic acid ester; LM985) was not recommended for phase II assessment because of drug associated acute hypotension and the fact that it appeared to function as a prodrug with rapid hydrolysis *in vivo* to FAA (Kerr *et al*., 1986).

Preclinical studies with FAA indicate that it is active against a broad spectrum of murine transplantable solid tumours which tend, on the whole, to be refractory to conventional cytotoxic agents. These included a range of colon adenocarcinomas, pancreatic ductal adenocarcinoma, mammary adenocarcinoma and Glasgow's osteosarcoma (Corbett *et al*., 1986; Zaharko *et al*., 1986).

Preclinical pharmacology (Zaharko *et al*., 1986) implied a plasma concentration threshold for activity and toxicity in mice and dogs, the plasma concentrations achieved in man in the phase I study were similar to those which were active in murine tumour models. Clinical phase I trials were conducted using both 1 h and 6 h infusions given weekly. At maximum tolerated doses – $6.4\,g\,m^{-2}$ over 1 h and $10\,g\,m^{-2}$ over 6 h – toxicity included hypotension, diarrhoea and intolerable warmth and flushing (Kerr *et al*., 1987). A phase II trial programme has been planned in Europe using both 1 h ($4.8\,g\,m^{-2}$) and 6 h ($8.6\,g\,m^{-2}$) schedules. We report here a phase II study of FAA, $8.6\,g\,m^{-2}$, administered by 6 h infusion to patients with advanced malignant melanoma and colorectal carcinoma. This was conducted under the auspices of the Cancer Research Campaign phase I/II trials committee.

### Patients and methods

Common eligibility criteria for these two disease specific protocols required that the patients have measurable disease, a performance status of 2 or less, life expectancy greater than 2 months, age under 75 years, appropriate haematological (haemoglobin >10 g %, WBC $>3{,}000\,mm^{-3}$ and platelets $>100{,}000\,mm^{-3}$) and biochemical (bilirubin $<20\,\mu mol\,l^{-1}$ and creatinine $<150\,\mu mol\,l^{-1}$) parameters, no radiotherapy during the preceding 4 weeks, no previous malignancy at other sites (except *in situ* cancer of cervix and adequately treated basal cell carcinoma of the skin) and no serious intercurrent non-malignant disease. The patients with malignant melanoma had not received prior chemotherapy while those with colorectal carcinoma had received no chemotherapy in the 4 weeks preceding treatment (6 weeks in the case of nitrosoureas and mitomycin C).

Flavone acetic acid was supplied by Lipha Lyonnaise Industrielle, dissolved in 1 litre of 0.9% saline and infused over 6 h. A total of 500 ml of 1.26% $NaHCO_3$ was infused over 1 h immediately before and after drug infusion in order to establish an alkaline diuresis. The drug was given every week for 6 consecutive weeks, haematological and biochemical parameters permitting. Blood pressure measurements were taken half-hourly during treatment and for an hour after the infusion finished.

Standard WHO response criteria were used where appropriate and in the absence of progressive disease or serious toxicity a further treatment course could be administered. WHO grades were not used for flushing, visual disturbance and hypotension. Arbitrary grades of I, mild, II, moderate and III, severe were substituted. Hypotension was graded as I if baseline <110 mm, up to 10% drop, or if baseline >110 mm, up to 20 mm drop; II if baseline <110 mm, 10–20% drop, or if baseline >110 mm, 20–40 mm drop; III if baseline <110 mm, more than 20% drop, or if baseline >110 mm, more than 40 mm drop. Patients in whom rapid disease progression occurred were considered eligible for response assessment if they had received a minimum of three doses at weekly intervals. Informed consent was obtained from all patients according to regulations determined by local ethical committees.

### Results

A total of 19 patients with colorectal carcinoma and 15 patients with malignant melanoma were entered in the trial. One patient with malignant melanoma (having received prior chemotherapy) was considered ineligible for the study. All but four of the eligible colorectal patients, who received only one or two courses due to rapid disease progression or drug toxicity, were evaluable for therapeutic response. The characteristics of evaluable patients are summarised in Tables I and II.

Treatment associated toxicity is shown in Table III. Overall, the toxicity as reported was mild. However, two patients were withdrawn from the study, one with drug associated hypotension and the other with grade IV haematological toxicity. The first patient had a drop in systolic blood pressure from 130 to 85 mmHg, 1 h after the second infusion commenced. The drug infusion was stopped and his blood pressure returned to normal after 1.5 h and no further drug treatment was given. The second patient had an episode of profound thrombocytopenia which developed shortly after her fifth cycle of FAA. Her platelet count fell to $11{,}000\,mm^{-3}$, she had spontaneous vaginal bleeding and required platelet transfusion. The patient recovered and subsequent investigations identified 1 gG and 1 gM antibodies to FAA on the patient's platelets. She had previously been treated in another study with a monoclonal antibody directed against carcino embryonic antigen and she had developed antimouse antibodies (Davis *et al*., 1988). In terms

Correspondence: D.J. Kerr.
Received 23 January 1989, and accepted in revised form 3 March 1989.

**Table I** Characteristics of evaluable colorectal patients

| *Characteristic* | *No. of patients* |
|---|---|
| Male/females | 10/5 |
| Median age (years) | 51 |
| Range | 42–71 |
| Median performance status | 1 |
| Range | 0–2 |
| Median no. of weekly courses given | 6 |
| Range | 4–9 |
| Previous surgery | 15 |
| Previous radiotherapy | 4 |
| Previous chemotherapy | 6 |
| Site of measurable disease | |
| Primary/regional recurrence/nodes | 9 |
| Liver | 12 |
| Lung | 3 |

**Table II** Characteristics of evaluable malignant melanoma patients

| *Characteristic* | *No. of patients* |
|---|---|
| Male/females | 5/9 |
| Median age (years) | 48 |
| Range | 28–69 |
| Median performance status | 0 |
| Range | 0–2 |
| Median no. of weekly courses given | 5 |
| Range | 3–6 |
| Previous surgery | 14 |
| Site of measurable disease | |
| Nodes/soft tissue/skin | 24 |
| Lung | 3 |
| Liver/intra-abdominal | 5 |
| Other | 2 |

**Table III** Patterns of toxicity for melanoma and colorectal carcinoma patients (usually consistent within patients and from week to week)

| | *Number of patients in grade* | | | |
|---|---|---|---|---|
| *Toxicity* | *I* | *II* | *III* | *IV* |
| Nausea and vomiting | 8 | 5 | 1 | – |
| Constipation | 3 | 1 | – | – |
| Diarrhoea | 6 | – | – | – |
| Hypotension[a] | 3 | 5 | – | – |
| Muscle pain | 7 | 3 | – | – |
| Flushing[a] | 3 | – | – | – |
| Visual disturbance[a] | 3 | – | – | – |
| Conscious state | 3 | – | – | – |
| Haematological | – | – | – | 1 |

Total number of evaluable patients = 29. WHO grades were used unless otherwise specified.

[a]These forms of toxicity were coded by an arbitrary scale defined in the Methods section.

of chronic toxicity, one female patient with colorectal cancer developed severe persistent postural hypotension after completing treatment. Investigations performed in the MRC Blood Pressure Unit, Western Infirmary, Glasgow, indicated that she had developed an autonomic neuropathy presumably related to FAA treatment (Lewis *et al.*, 1988). She had good symptomatic control with fludriocortisone.

In terms of disease response, 13 of the melanoma patients progressed on treatment and one had stable disease at the end of treatment. Nine of the evaluable colorectal patients progressed on treatment and six had stable disease at the end of treatment.

### Discussion

Despite promising preclinical activity, FAA (8.6 g m$^{-2}$ over 6 h) has no demonstrable clinical activity in advanced malignant melanoma and colorectal carcinoma. This result is similar to that reported by the Early Clinical Trials Group of the EORTC who found no activity in phase II trials in four tumour types using 4.8 g m$^{-2}$ over 1 h (Kaye *et al.*, 1989). In the phase I trial, the pharmacokinetic profiles indicated that 'effective drug exposure', as assessed by the area under the plasma concentration time curve $>100\,\mu g\,ml^{-1}$, was approximately 50% greater for the 6 h infusion. Despite this apparent pharmacokinetic advantage, the 6 h infusion is also clinically inactive.

The toxicity associated with the phase II trials was similar to that predicted by the phase I trial. One patient suffered persistent toxicity in the form of postural hypotension caused by autonomic neuropathy, presumably related to treatment with FAA (Lewis *et al.*, 1988) but no other 'late' toxicity has been seen.

The mechanism of action of FAA is unknown. There is only minimal damage to DNA, as assessed by alkaline elution (Bissery *et al.*, 1988). However, there is some evidence to suggest the FAA can induce haemorrhagic necrosis in murine colonic tumours (Smith *et al.*, 1987) and decrease blood flow in subcutaneously implanted Glasgow's osteosarcoma as demonstrated by $^{31}P$ nuclear magnetic response spectroscopy (Evelhoch *et al.*, 1988). In view of the clinical effects of FAA on blood pressure it is tantalising to hypothesise that differences in the regulation of tumour and systemic blood flow, between mouse and man, could contribute to FAA's inactivity clinically. In addition, FAA systemically augments natural killer cell activity in normal and tumour bearing mice and in human cancer patients, possibly by induction of $\alpha$ and $\beta$ interferon (Horning *et al.*, 1988), but the clinical relevance of these observations is unknown.

Although there are differences in plasma binding of FAA comparing mouse and man, the plasma concentrations achieved at the recommended phase II doses are similar to those active in murine tumour models. FAA is extensively metabolised to glucoronides in patients, but not mice, and the drug has a higher relative plasma clearance in humans (J. Cummings, personal communication). It would be interesting to compare tumour FAA concentrations in mouse and man, but it seems more likely that its clinical inactivity may relate to differences in its biological effects in murine and human tumour systems.

This study was conducted on behalf of the Cancer Research Campaign Phase II Clinical Trials Committee and supported by Lipha Lyonnaise Industrielle Ltd. D.J.K. is supported by the CRC and would like to thank Fiona Conway for typing the manuscript.

### References

BISSERY, M.-C., CORBETT, T.H., CHABOT, G.G., CRISSMAN, J.D., YOST, C. & VALERIOTE, F. (1988). Flavone acetic acid (NSC 347512)-induced DNA damage in Glasgow osteogenic sarcoma *in vivo*. *Cancer Res.*, **48,** 1279.

CORBETT, T.H., BISSERY, M.-C., WOZNIAK, A. and 5 others (1986). Activity of flavone acetic acid (NSC-347512) against solid tumours of mice. *Invest. New Drugs*, **4,** 207.

EVELHOCH, J.L., BISSERY, M.-C., CHABOT, G. and 4 others (1988). Flavone acetic acid (NSC-347512)-induced modulation of murine tumour physiology monitored by *in vivo* nuclear magnetic response spectroscopy. *Cancer Res.*, **48,** 4749.

DAVIS, H.P., NEWLANDS, E.S., ALLAIN, T. & HEDGE, U. (1988). Immune thrombocytopenia caused by flavone-8-acetic acid. *Lancet*, **i,** 412.

HORNING, R.L., YOUNG, H.A., URBA, W.J. & WILTROUT, R.H. (1988). Immunomodulation of natural killer cell activity by flavone acetic acid: occurrence via induction of interferon β. *J. Natl Cancer Inst.*, **80**, 1226.

KAYE, S.B., CLAVEL, M., DODION, P. and 5 others (1989). Phase II trials of flavone acetic acid in patients with cancers of the breast, colon, lung, head and neck and melanoma. *Invest. New Drugs* (in the press).

KERR, D.J., KAYE, S.B., CASSIDY, J. and 8 others (1987). Phase I and pharmacokinetic study of flavone acetic acid. *Cancer Res.*, **47**, 6776.

KERR, D.J., KAYE, S.B., GRAHAM, J. and 8 others (1986). Phase I and pharmacokinetic study of LM985 (flavone acetic acid ester). *Cancer Res.*, **46**, 3142.

LEWIS, C., JARDINE, A., RANKIN, E.M. & KAYE, S.B. (1988). Autonomic neuropathy following treatment with flavone acetic acid. *Eur. J. Cancer Clin. Oncol.* (in the press).

SMITH, G.P., CALVELEY, S.B., SMITH, M.J. & BAGULEY, B.C. (1987). Flavone acetic acid (NSC 347512) induces haemorrhagic necrosis of mouse colon 26 and 38 tumours. *Eur. J. Cancer Clin. Oncol.*, **23**, 1209.

ZAHARKO, D.S., GRIESHABER, C.K., PLOWMAN, J. & CRADOCK, J.C. (1986). Therapeutic and pharmacokinetic relationships of flavone acetic acid: an agent with activity against solid tumours. *Cancer Treat. Rep.*, **70**, 1415.